# Dengue virus serological markers among potential blood donors: an evidence of asymptomatic dengue virus transmission in Cameroon

**DOI:** 10.11604/pamj.2020.36.185.22128

**Published:** 2020-07-14

**Authors:** Salomon Bonsi Tchuandom, Abel Lissom, Ghislaine Haverie Mimfoumou Ateba, Thibau Flaurant Tchouangueu, Constantin Tchakounte, Agbor Rolland Ayuk, Etienne Philemon Atabonkeng, Ankiambom Innocent Ngong, Godwin Nchinda, Jules-Roger Kuiate

**Affiliations:** 1 Department of Biochemistry, University of Dschang, Dschang, Cameroon,; 2 Public School of Medical Laboratory Technicians, Yaoundé, Cameroon,; 3 Laboratory of Vaccinology/Biobanking, CIRCB, Melen-Yaoundé, Cameroon,; 4 Department of Animal Biology and Physiology, University of Yaoundé 1, Yaoundé, Cameroon,; 5 Blood transfusion service, Yaounde Jamot Hospital, Yaoundé, Cameroon,; 6 National Public Health Laboratory, Yaoundé, Cameroon

**Keywords:** Dengue fever, potential blood donors, seroprevalence, Cameroon

## Abstract

**Introduction:**

the risk of dengue virus or its antibodies which can be transmitted through blood transfusion by asymptomatic individuals infected, has been a major concern all over the world. Dengue is an endemic disease in sub-Saharan Africa, particularly in Cameroon. The purpose of this study is to determine the frequency of dengue virus (DENV) infection among potential blood donors at Yaounde Jamot Hospital.

**Methods:**

serum samples were collected from 310 potential adult blood donors aged 18-57 years, who signed a written informed consent and completed the questionnaire between March 2019 and August 2019. This serum is used to screen for the presence of serological markers of DENV infection (NS1, IgM and IgG) using immunochromatographic tests (Zhuhai Encode Medical Engineering Co., Ltd, China). IgM/IgG positive samples were confirmed by enzyme-linked immunosorbent assays (ELISA).

**Results:**

the overall prevalence was 24.8% among potential blood donors were subdivided as follows: 4.5% (14/310), 12.3% (38/310) and 6.1% (19/310) showed mono-positivity to DENV-NS1 antigen, anti-DENV IgM and anti-DENV IgG antibodies respectively. 1.9% (6/310) of potential blood donors showed dual positivity to anti-DENV IgM antibodies and anti-DENV IgG antibodies. The presence of DENV-NS1 antigen show asymptomatic viremia of dengue at the time of donation, while the presence of IgG antibodies reflects the high endemicity of dengue disease in the city of Yaoundé.

**Conclusion:**

these findings demonstrate the high level of risk of the DENV transmission among potential blood donors to needy recipients, underscoring the importance of establishing dengue fever blood screening in different services and blood collection units in Cameroon to improve safety transfusion and control the dissemination of the DENV.

## Introduction

Dengue fever is the largest arbovirus in the world and it is endemic in more than 128 countries, with roughly 3.5-4 billion people, exposing them to the dengue virus (DENV) infection, with over 50.000 deaths per year worldwide [1]. Blood transfusion represents an important neglected form of DENV transmission and endemicity in the community beyond the standard mode of transmission by mosquito bites of the *Aedes* genus [2-8]. These mosquitoes are present in all regions of Cameroon [9]. DENV infection is caused by a single-stranded RNA virus with four serotypes (DENV-1, DENV-2, DENV-3 and DENV-4) [7]. Up to 50-85% of people infected with DENV remain asymptomatic and these people represent an important reservoir of DENV and anti-DENV IgM/IgG antibodies [10,11]. Studies conducted worldwide have shown the presence of DENV-NS1 antigen or anti-DENV IgM/IgG antibodies in blood donors [2, 3, 12, 13]. The data of the dengue in blood donors are scarce in sub-Saharan Africa especially in Cameroon. In 2018, approximately 94873 bags of blood were collected across Cameroon. This is 23.7% coverage in blood bags, whose annual needs are estimated at 400,000 safe blood bags [14]. In Cameroon, the DENV was first reported in Mora in 1984 on febrile patients [15]. To date, dengue has been found in several cities in Cameroon among asymptomatic people [16]. Dengue remains an endemic disease in Cameroon [17] although there was a recent epidemic in Kribi (South Cameroon) in 2018 [18].

Further epidemics can have far reaching consequences if blood bags are contaminated with DENV or its antibodies remains. Causes include migrants who came in sub-Saharan African countries like Nigeria with dengue epidemics and even the persons running from conflicting areas or towns in Cameroon may come during the viraemic at the asymptomatic phase of dengue to donate remunerated blood [19, 20]. The clinical picture of symptomatic dengue fever ranges from relatively mild to severe dengue fever that can be induced by DENV or by the presence of antibodies in blood recipient [6, 21]. Transfusion of blood from donors having partially or neutralizing anti-DENV antibodies may increase the immunological susceptibility of recipients [3, 4, 8, 22], have been proposed to authors including social vulnerability with recipient/vector prominence [23]. These recipients have a higher risk of developing severe forms of dengue fever if they are infected within months of transfusion with another DENV serotype [24], especially in an environment where all the four serotypes circulate like in Cameroon [22]. Although systematic tests are always performed, dengue screening is not performed among potential blood donors. The purpose of this study is to investigate serological markers of DENV infection on potential blood donors at Yaounde Jamot Hospital in order to improve safety transfusion in Cameroon's blood donor services.

## Methods

**Study and research design:** this was a cross sectional descriptive study conducted at the blood transfusion service of the Yaounde Jamot Hospital, which is one of the referral centres for blood in the capital city of Cameroon. Data was collected between March and August 2019 from potential donors negative to systematic infectious markers such as hepatitis B surface antigen, anti-hepatitis C virus, human immunodeficiency virus and anti-treponema pallidum antibodies. The concept of the study was explained to the donors and their writings informed consent sought according to the policy set up by the ministry of public health of Cameroon. Demographic data such as: name, address, sex, age, categories of donation and professional mobility were appropriately collected with a designed questionnaire.

**Sample size determination:** the formula n = Z^2^PQ / d^2^was used to derive the desired sample size, where n is the sample size, P the prevalence, Q is 1-P, Z is the statistic corresponding confidence level (1.96), d is precision (0.05) [25]. A prevalence of 24.2% in asymptomatic person was used, representing maximum uncertainty [16]. In conclusion, the estimated sample size was 282.

**Sample collection and processing:** about five millilitres of blood samples were collected by venepuncture from eligible donors into EDTA tube. Whole blood samples were centrifuged for 5 minutes at 3500 rpm to obtain plasma. Plasma fractions were aliquoted into plain cryovial tubes. The samples were then stored at -20^o^C for subsequent analyses.

**Laboratory test for dengue infection:** each plasma was analysed for the detection of NS1 antigen and for the detection of IgM/IgG antibodies using dengue IgG/IgM/NS1 combo rapid test (Zhuhai Encode Medical Engineering Co.,Ltd). The interpretation of the RDTs results was done in conformity with the manufacturer´s instructions. IgM and IgG antibodies against DENV were confirmed using an in-house indirect ELISA assay, as previously described [7, 26].

**Statistical analysis:** data obtained from the questionnaires were entered and managed in Excel spreadsheets. All data were analysed using Graph Prism (Graph pad 6.0, San Diego, USA). We calculated the overall seropositivity rates. The Fisher exact and Chi-square tests were used to evaluate associations and statistical significance of the distribution of the outcome among the different variables. Using bivariable logistic regression, we examined the impact of being stable at the jobsite on the odds of DENV seropositivity.

**Ethics approval and consent to participate:** ethical clearance for this study was obtained from the Cameroon National Ethics Committee for Research on Human Subjects (Ethical Clearance number N°199/CRERSH/2019). Written consent form of each participant in this study was sought and obtained after explaining the risks during venepuncture, the benefits and the right to refuse participation or to decline any question item. Demographic data such as: name, address, sex, age, type of donor and risks factors were collected with a designed questionnaire.

## Results

**Study population:** a total of 310 blood donors were recruited in this study. As shown in Table 1, 91.6% (284) were men versus 8.4% (26) female donors. The mean age (± SD) of the total population was 29.4 ± 7.8 years, with likely mean ages between both sexes (P = 0.968). Globally, donors of the age groups ranging from 18-25 years and 16-33 years were the most represented, even though the difference of age group distribution was not significant (P = 0.9721). During recruitment, donors were divided into three groups including familial donors (who gave blood for a family member), voluntary donors (who gave blood to help a patient) and replacement donors (who freely gave blood for personal motivations). It was shown that there were more familial donors (58.1%) than the voluntary (22.9%) and the replacement (19%), despite the homogenous distribution (P = 0.1638) among male and female donors. As for professional occupations, the overall proportion of donors who were stable at their workplace (74.2%) seemed greater than hawkers donors during their duties (25.8%), even if they were distributed between both sexes (P = 0.4919). The proportion of donors from secondary school (45.5%) was almost similar to that of high school (54.5%). However, there was a prominent proportion of male donors from secondary level compared to females, subsequently, an inverse distribution was noticed from donors from higher level (P = 0.0223) (Table 1).

**Table 1:** general characteristic of study population

	Men (n=284)	Women (n=26)	Total (n=310)	P-value
**Ages (years)a**	**29,4 ± 7,9**	**29,5 ±7,3**		**0,968**
**Ages ranges % (n)**				
18-25	92 (104)	8 (9)	36,4 (113)	0,9721
26-33	91,2 (103)	8,8 (10)	36,4 (113)	
34-41	89,7 (52)	10,3 (6)	18,7 (58)	
42-49	100 (21)	0 (0)	6,9 (21)	
50-57	80 (4)	20 (1)	1,6 (5)	
**Type of donors % (n)**				
Voluntary	20,1 (57)	7,7 (2)	19 (59)	0,1638
Family	58,1 (165)	57,7 (15)	58,1 (180)	
Replacement	21,8 (62)	34,6 (9)	22,9 (71)	
**Type of activity % (n)**				
Hawker	26,4 (75)	19,2 (5)	25,8 (80)	0,4919
Non-mobile	73,6 (209)	80,8 (21)	74,2 (230)	
School level % (n)				
Secondary	47,5 (135)	23,1 (6)	45,5 (141)	0,0223
High school	52,5 (149)	76,9 (20)	54,5 (169)	

a: the ages were expressed in mean ± standard deviation (SD); For each characteristic the proportions were compared between men and women; n: number of donors

**Overall prevalence of dengue virus infection among study population groups:** overall, 24.8% (77/310) of donors were positive to at least a DENV infection marker. The results of Table 2 showed the distribution of DENV positive and negative donors among the groups of the study population. In this table, DENV infection showed no significant association with either the sex, educational level and the donors´ category. However, the increased of DENV prevalence among potential blood donors was significant depending (P = 0.0026) on the type of activity of these donors at their workplace and the odd of dengue infection (OR: 0.348; CI [0.169 - 0.715]). In fact, individuals having non-mobile jobs (29.1%) are more prevalent to DENV infection compared to hawkers (12.5%). Although the difference was not significant (P = 0.7387), individuals aged 42-49 years had the highest dengue prevalence (33.3%) of all age groups (Table 2).

**Table 2:** prevalence of dengue virus infection among blood donors according to sociodemographic characteristics

	Dengue serology			
	Positive (n=77)	Negative (n=233)	total (n=310)	p-value
**% (n)**	**24,8 (77)**	**75,2 (233)**	**310**	**NT**
**Ages**	**29,5±7,5**	**29,4 ±7,9**	**29,4± 7,8**	**0,92**
**Ages ranges % (n)**				
18-25	24,8 (28)	75,2 (85)	36,4 (113)	0,7387
26-33	26,5 (30)	73,5 (83)	36,4 (113)	
34-41	20,7 (12)	79,3 (46)	18,7 (58)	
42-49	33,3 (7)	66,7 (14)	6,9 (21)	
50-57	0 (0)	100 (5)	1,6 (5)	
**Sex % (n)**				
Men	24,6 (70)	75,4 (214)	91,6 (284)	0,8138
Women	26,9 (7)	73,1 (19)	8,4 (26)	
**Type of donors % (n)**				
Voluntary	22 (13)	78 (46)	19 (59)	0,12 (4,23)
Family	28,9 (52)	71,1 (128)	58,1 (180)	
Replacement	16,9 (12)	83,1 (59)	22,9 (71)	
**Type of activity % (n)**				
Hawker	12,5 (10)	87,5 (70)	25,8 (80)	0,0026a
Non-mobile	29,1 (67)	70,9 (163)	74,2 (230)	
**School level % (n)**				
Secondary	22,7 (32)	77,3 (109)	45,5 (141)	0,43
High school	26,6 (45)	73,4 (124)	54,5 (169)	

a: Odds ratio was 0.348, with CI [0.169 - 0.715]; For each characteristic the proportions were compared between men and women; NT: Not tested; n: number of donors

**Figure 1:**
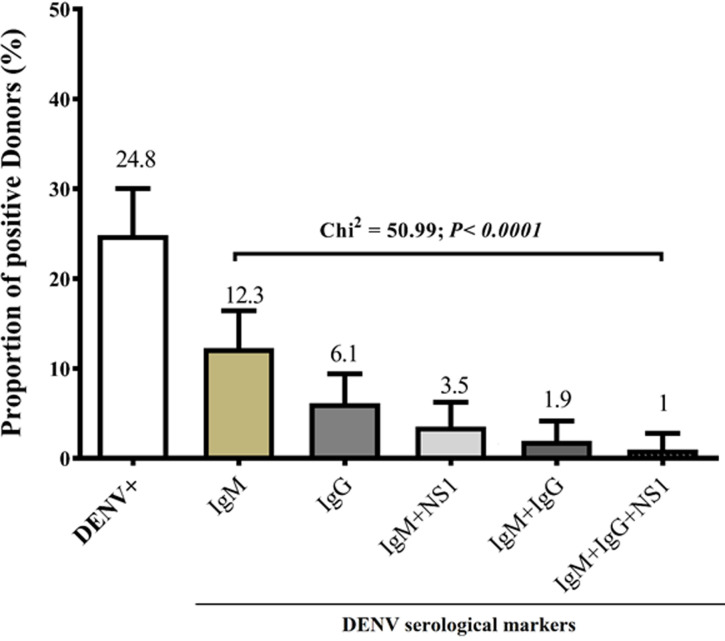
distribution of donors with respect to the serological markers of dengue virus

**Serological markers of dengue virus infection in blood donors:** the prevalence of serological markers of dengue within this study population were determined as in Figure 1 (The proportions of serological markers of dengue were determined in the total population. The data were expressed in percentage with confidence interval (CI). DENV+: dengue positive individuals. IgM: group of individuals only positive to IgM antibody specific to DENV. IgG: group of individuals only positive to IgG antibody specific to DENV. IgM+NS1: group of individuals positive to NS1 dengue virus protein and IgM antibody specific to DENV; IgM + IgG: group of individuals positive to IgM and IgG antibodies specific to DENV IgM + IgG + NS1: individuals positive to NS1 dengue virus protein, IgM and IgG antibody specific to DENV) and Table 3. In Figure 1, potential blood donors who were positive for DENV-NS1 antigen (n = 14/310, 4.8%) were subdivided as follows: 3.5% (11/310) and 1% (3/310) of blood donors showed DENV-NS1 antigen plus anti-DENV IgM antibodies and DENV-NS1 antigen plus anti-DENV IgM/IgG respectively. Various proportions of anti-DENV antibodies were found in blood donors, with 12.3% (38/310) and 6.1% (19/310) for anti-DENV IgM and IgG antibodies respectively and 1.9% (6/310) dual positivity to anti-DENV IgM/IgG antibodies. A significant difference was shown when comparing the proportions of DENV markers (Chi^2^= 50.99; P < 0.0001). Nevertheless, no significant association was shown when comparing proportions of DENV serological markers among blood donors of this study population (Table 3).

**Table 3:** distribution of dengue serological markers with respect to the blood donor characteristics

	NS1	IgM	IgG	IgM+IgG	p-value
**Sex % (n)**					
Men (n= 284)	15.7 (11)	52.9 (37)	22.8 (16)	8.6 (6)	0.1055
Women (n= 26)	42.9 (3)	14.2 (1)	42.9 (3)	0	
**Type of donors % (n)**					
Voluntary (n=59)	23.1 (3)	46.1 (6)	15.4 (2)	15.4 (2)	0.7009
Family (n=180)	17.2 (9)	48.1 (25)	25 (13)	9.6 (5)	
Replacement (n=71)	16.9 (2)	33.3 (4)	33.3 (4)	16.8 (2)	
**Type of activity % (n)**					
Hawker (n= 80)	25 (4)	37.5 (6)	25 (4)	12.5 (2)	0.9107
Non-mobile (n= 230)	17 (10)	50.8 (30)	25.4 (15)	6.8 (4)	

n: number of donors; NS1: donor who were positive to NS1 protein with other antibodies

## Discussion

In safety transfusion, a zero-risk approach does not exist. Post-donation accidents still persist despite all measures taken. This study was designed to screen for NS1 antigen and anti-DENV IgM/IgG antibodies to DENV in potential blood donors from whom blood bags should be collected to save lives. This study is probably one of the first to address the issue of asymptomatic dengue among potential blood donors in sub-Saharan Africa and more especially in Cameroon, which could easily explain the paucity of the literature on the subject. This work focused on a sample of 310 eligible blood donors with an average age of 29.4 ± 7.8 years, the extremes being 18 years and 57 years. These results are in line with those of Chaudhari *et al*. who found an average age of 28.2 years ± 7.2 [27]. The findings of this study show that 91.6% of blood donors were male. This result is consistent with that found by Ashshi *et al*. (100%) in the studies conducted in Saudi Arabia involving eligible blood donors [28]. Blood donors in Saudi Arabia are essentially male because of their culture. The male majority is due to blood donation criteria restricting female contributions due to, menstruation, pregnancy and breastfeeding and other female associated risk of post blood donation in this group [29]. The study found that 66.8% of blood donors were between 18-33 years of age. This outcome is similar to the one found in North India in 2015 [3]. Voluntary blood donors in this study were not many (19.0%) compared to familial donors (58.1%) or replacement donors (22.9%).

This contrasts with data from the National Blood Transfusion Program, which in 2017 showed that 10% of blood donors were voluntary while 90% of donors were family replacement donors [30]. In fact, voluntary donors are a safe source of blood and since the last conference of voluntary blood donors in Cameroon, the government, through the Ministry of Public Health, has provided extensive technical, financial and material support to raise awareness amongst voluntary blood donors. Blood donors from academia were statistically the most important. This finding could be explained on the basis of the analysis by the educational mobility analysis showing that current generations are better educated than previous ones (regardless of gender) about the importance of blood donation as a drug, far from the taboos embodied in the population in Cameroon [31]. Most blood donors had a job that kept them stable (74.2%) unlike donors with a mobile activity. Indeed, the employment structure based on the sector is dominated by the primary sector in rural areas and the tertiary sector in urban areas. Jobs in the tertiary sector do not require some degree of movement for people who perform them [31]. Dias *et al*. found that 70% of DENV were in the bloodstream 7 days after infection on asymptomatic persons [32]. The existence of the seroconversion phase suggests the need to find serological markers of the dengue fever virus among blood donors. The overall prevalence of dengue fever among blood donors was 24.8%.

Our result is lower than prevalence shown in 2017 among asymptomatic blood donors (94.2%) in western India [33]. This disparity can be explained by the existence of serious dengue epidemics in India, unlike Cameroon where dengue fever is endemic [17]. Dengue fever was highly prevalent (29.1%) among blood donors with a stationary profession-related activity as contrasted with those with a mobile activity (12.5%). This observation corroborates with the dynamism of dengue vectors that sting less when people are in motion [34]. Dengue fever incidence in this study was 4.5% (14/310) considering the presence of DENV-NS1 antigen in blood donors. This value is similar to findings found by Ashshi *et al*. in Saudi Arabia that showed a 5.3% incidence of DENV-NS1 antigen in eligible blood donors [28]. However, this is inconsistent with the results found by Kulkarni *et al*. [33] in India, Rooks *et al*. [35] in Australia and Mangwana *et al*. [3] in India, where DENV-NS1 antigen incidences of 0.64%, 0.0% and 0.0% respectively in eligible blood donors are shown. Biological diagnosis of DENV-NS1 antigen alone or in combination with anti-DENV IgM/IgG antibodies in a patient reveals acute dengue fever and displays a viraemic phase of DENV infection in these patients. Also, NS1 antigen can be detected during this phase that can last up to 9 days [28, 36]. Seroprevalences of anti-DENV IgM and IgG antibodies were 12.3% (38/310) and 6.1% (19/310) respectively. These contrasts with studies done by Ashshi *et al*. and Kulkarni *et al*. which found 5.5% and 6.4% for positive anti-DENV IgM antibodies, 38.9% and 87.0% for positive anti-DENV IgG antibodies cases respectively [28, 33].

In addition, the co-occurrence of anti-DENV IgM/IgG antibodies in the same plasma resulted in a seroprevalence of 1.9% (6/310). According to WHO recommendations [36], a case of dengue fever is considered as primary in the absence of anti-DENV IgG antibodies. The seropositivity of anti-DENV-IgM antibodies in blood donors indicates the carrier stage of DENV infection [22, 37-39]. Noticeably, the DENV carrier stage in the local population have greatly increased from 0.0% [16] in 2006 to 12.5% as investigation were carried out in the current study. This disparity might be due to the method of testing. However, anti-DENV-IgG antibodies demonstrates the endemicity of dengue fever in Cameroon [7, 17]. These findings show that donors are believed to come from households with characteristics favourable to the distribution of DENV, vectors, circulation and/or introduction of one or more serotypes, several favourable environmental parameters (temperature and precipitation affect the demography and habits of vectors), increasing international and national transport, poverty and the lack of such a programme on dengue fever. Earlier studies have been discussed in Cameroon including the existence of all the four serotypes, we could have investigated to determine DENV serotypes among donors as well as performing plaque reduction neutralization test to exclude cross-reaction with other flaviviruses genus.

## Conclusion

The endemic character in Cameroon makes potential blood donors expose to the dengue virus. This shows the presence of DENV in blood donors. Thus, the government must integrate dengue virus diagnosis into routine blood transfusion tests, periodically carry out a large-scale vector control campaign and set up a vast program for dengue fever.

### What is known about this topic

- Dengue virus infection is 80% asymptomatic;
- In Asia, dengue virus was found to be positive among blood donors;
- Several localities in Cameroon were highly prevalent to dengue virus infection.

### What this study adds

- Dengue virus infection among potential blood donors was highly prevalent in Cameroon;
- Positivity to dengue virus infection must not be associated to the exposure to mosquito bites, but a result of a silent transmission in the Cameroonian population.
